# Critical roles of functional molecule metabolites

**DOI:** 10.3389/fmolb.2023.1119588

**Published:** 2023-02-15

**Authors:** Peng Liu, Ying Cai, Shi Qiu, Qiang Yang, Yiqiang Xie, Aihua Zhang

**Affiliations:** ^1^ International Advanced Functional Omics Platform, Scientific Experiment Center, Hainan Medical University, Haikou, China; ^2^ Experimental Center and Graduate School, Heilongjiang University of Chinese Medicine, Harbin, China

**Keywords:** metabolites, metabolomics, genotype, phenotype, function, functional metabolomics, metabolism, diagnosis

Recently, four studies on the potential value of small-molecule metabolites by high-throughput functional metabolomics were published in *Nature Medicine* (Buergel et al., 2022; Chen et al., 2022; Kachroo et al., 2022; Surendran et al., 2022). These studies have shown that metabolic profiling of small molecules offers invaluable insights into the function of candidate metabolites, leading to better diagnosis, prognosis, and therapeutic effects. These four publications have elucidated how the metabolite biosignatures as attractive biomarkers from human biofluids provide a link between the genotype, environment, and phenotype.

**FIGURE 1:**
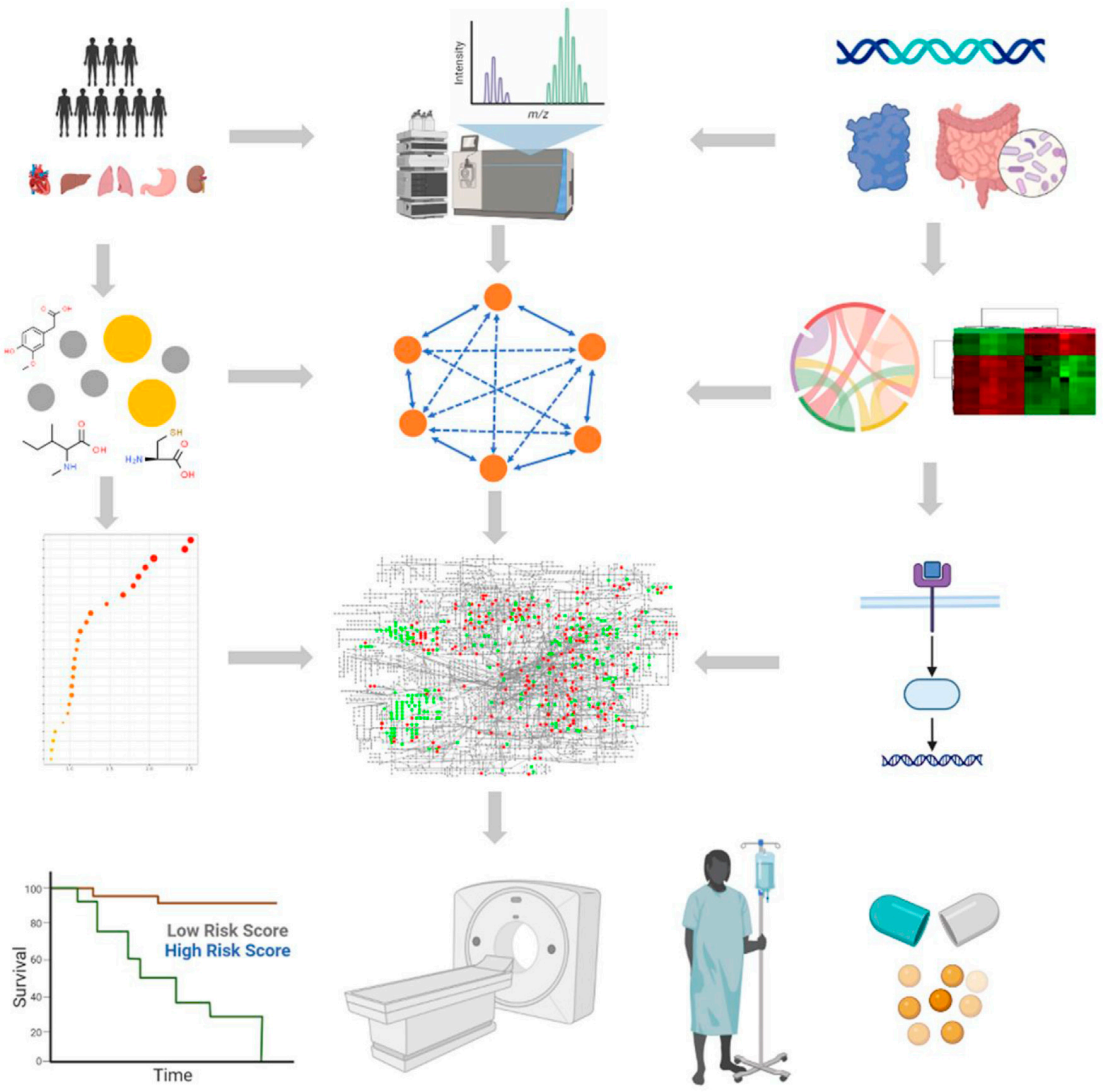
Brief workflow of small-molecule metabolite-based functional metabolomics. The first stage involves experimental design, followed by the election of biological subjects, sample collection, preparation, and metabolite extraction. Metabolites are detected using specific detection techniques; data pre-processing is used to pre-process raw signals to produce data in a suitable format for subsequent statistical analysis; then, data normalization is used to reduce the system and technical bias; next, small-molecule metabolites are filtered and quantified for significant biomarkers of interest; statistical analyses and univariate and multivariate statistical analyses are used to identify significantly expressed metabolites; and finally, data are interpreted through biomarker discovery and functional interpretation. Multi-omics (genome, transcriptome, proteome, metabolome, and microbiome) data are collected from patients and integrated to identify personalized functional signatures using complex and comprehensive network analysis. Next, the significantly expressed metabolites are subsequently linked to the biological context by enrichment and pathway analysis. Applications of small-molecule candidate metabolites for biomarker discovery, disease diagnosis, prognosis, and treatments in clinical studies. Figure was drawn using BioRender and MetaboAnalyst.

Metabolic abnormalities lead to the accumulation or deficiency of small-molecule metabolites, which are related to phenotypic variation, can reveal the pathological mechanisms of various diseases and decipher therapeutic potential (Bar et al., 2020). Functional metabolomics is defined as a research strategy for discovering differential metabolites from large metabolome that comprehensively uses molecular and cell biology, bioinformatics, metagenome, and transcriptome to explore the biological functions of metabolites and related physiological as well as pathological significance (Figure 1). It provides an innovative approach to answering phenotype-related questions distinctly altered in diseases, elucidating the biochemical functions, and delineating the associated mechanisms implicated in the dysregulated metabolism in patients within clinical settings.

High-throughput metabolomics technology has been used to identify phenotypic variation arising in the human plasma metabolome that is influenced by dietary, genetic, and microbial factors. Chen et al. (2022) first evaluated a total of 1,183 plasma metabolites in the Dutch cohort and then conducted an interaction analysis to assess the critical effect of diet, intestinal microbiome, and genetics on human metabolism regulation and showed that 610, 85, and 38 metabolites were significantly related to each type of factors (diet, genetics, or microbiome), respectively. These metabolites show key links between the genotype and environment and provide a closer image of the final phenotype. The authors then used the plasma metabolome to explore and predict diet quality and illustrated the relationship between human metabolism and diet. Notably, they observed a significant correlation between 1-methylhistidine and meat and fish intake, as well as lower levels of 1-methylhistamine in vegetarians. They combined plasma metabolites and genetic association to provide functional insight into disease etiology and found that 5-acetamido-6-formylamino-3-methyluracil has the strongest association with the *N-acetyltransferase 2* gene, which is linked to the risk of bladder cancer.

Since small-molecule metabolites are closely linked to the phenotype, they govern the modulation of its function, thus reflecting the interactions with the environment. The gut microbiota generates active metabolites which enter into the circulation system and regulate host metabolism responses and metabolic disorders *in vivo* that affect systemic health. The authors further observed the potential causal relationship to assess the contribution of the microbiome to the plasma metabolome and found that the increased level of adenosylcobalamin biosynthesis was related to the reduced level of 5-hydroxytryptophan that is associated with Parkinson’s disease; the plasma level of hydrogen sulfite was related to *Eubacterium rectale*, which interfered with cardiovascular function, nervous system, and inflammatory process (Chen et al., 2022). In short, functional relationship analysis among diet, genetics, and microorganisms with small-molecule metabolites showed that these factors could influence the metabolic phenotypes of diseases, improve our understanding of the pathological mechanisms of complex diseases, and also provide preventive and precise therapeutic strategies.

In order to verify the attributions of global metabolites, Buergel et al. (2022) first established the metabolic profile as a disease assay to simultaneously identify multiple disease risks and found that the predictive value of marker metabolites for eight common diseases exceeded the conventional clinical variables. Most remarkably, the authors revealed a significant correlation between creatinine and AAA, GlycA with lung cancer and COPD, and glucose and T2D. Level changes in these metabolites will improve their specificity for patient diagnosis, risk prediction, and prognosis. Buergel’s research group demonstrated that small-molecule phenotype signatures provide effective avenues for a better understanding of pathological functional states and molecular characteristics of diseases and for developing early biomarkers to improve patient prevention and management in clinics. Kachroo et al. (2022) first utilized metabolomic profiling and revealed a significant association between inhaled corticosteroid therapy and adrenal insufficiency symptoms in 14,000 asthmatic patients. The authors discovered that the levels of 17 endogenous steroid metabolites in asthmatic patients were significantly reduced, and further evidence showed that this reduction was closely related to inhaled corticosteroid therapy. This study showed that the great potential of metabolic profiling can provide comprehensive and accurate pictures of phenotypes related to various diseases or drug monitoring. A snapshot of human metabolism was also revealed by Surendran et al. (2022), and untargeted high-throughput metabolomics identified a total of 2,599 metabolite-specific associations in 19,994 individuals and provided insights into metabolite function and drug effects.

Taken together, these groundbreaking studies provide substantial support for the use of high-throughput metabolomics for identifying small-molecule metabolites and improving disease diagnosis, prognosis, and treatment response for ameliorating the quality of life in patients. Small-molecule metabolites showed a correlation with the functional status of a biological system. They pave the way for the understanding of metabolic alterations under different conditions to design the intervention strategy for manipulating small-molecule metabolites. These clinical applications also provide excellent examples to illustrate how the metabolites could facilitate the prediction of diseases or treatment, better explore potential molecular mechanisms within disease progression, and enable precision-targeted therapies.

Advancements in high-throughput metabolomics technologies have demonstrated the potential value of the investigation of metabolite signatures and allowed the researcher to establish comprehensive metabolic profiling to provide insights into metabolic functions. However, several challenges need to be addressed in future research. Endogenous metabolites may participate in all kinds of metabolic processes, thus affecting human health, but it is still difficult to detect them quickly while maintaining their metabolic status. Future research should define and continue to focus on the functional correlation with disease pathogenesis. Nevertheless, method standardization and processes should be reflected in each stage, and the predictive ability and diagnostic value of the established metabolic profiles need to be further validated in a large number of cohort studies. In the future, advanced artificial intelligence, computational algorithms, and big data mining analytics will be urgently needed to improve coverage of low-abundance metabolites for clinical validity. From clinical to experimental and from experimental to clinical, multiple cycles of validation can achieve the optimal model to thoroughly understand the functional significance of small-molecule metabolites.
